# Time Difference of Arrival on Contrast-Enhanced Ultrasound in Distinguishing Benign Inflammation From Malignant Peripheral Pulmonary Lesions

**DOI:** 10.3389/fonc.2020.578884

**Published:** 2020-11-12

**Authors:** Min Tang, Qianrong Xie, Jiasi Wang, Xiaoyu Zhai, Hong Lin, Xiaoxue Zheng, Guoli Wei, Yan Tang, Fanwei Zeng, Yanpeng Chu, Jianqiong Song, Jianqiang Cai, Fanxin Zeng

**Affiliations:** ^1^ Department of Ultrasound Imaging, Dazhou Central Hospital, Dazhou, China; ^2^ Department of Clinical Research Center, Dazhou Central Hospital, Dazhou, China; ^3^ Key Laboratory of Carcinogenesis and Translational Research (Ministry of Education/Beijing), Department of Thoracic Medical Oncology, Peking University Cancer Hospital and Institute, Beijing, China; ^4^ Department of Public Health Information, Sichuan Center for Disease Control and Prevention, Chengdu, China; ^5^ Department of Hepatobiliary Surgery, National Cancer Center/Cancer Hospital, Chinese Academy of Medical Sciences and Peking Union Medical College, Beijing, China

**Keywords:** contrast-enhanced ultrasound, peripheral pulmonary lesions, time difference of arrival, benign inflammation lesions, malignant lesions****

## Abstract

**Introduction:**

Worldwide, the incidence and mortality of lung cancer are at the highest levels, and the most lesions are located in the lung periphery. Despite extensive screening and diagnosis, the pathologic types of peripheral pulmonary lesions (PPLs) are difficult to diagnose by noninvasive examination. This study aimed to identify a novel index—time difference of arrival (TDOA)—to discriminate between benign inflammation and malignant PPLs.

**Methods:**

Using contrast-enhanced ultrasound (CEUS), we retrospectively analyzed 96 patients with PPLs who had undergone biopsy to confirm the pathologic types. All data were collected from Dazhou Central Hospital between December 2012 and July 2019. The parameters of CEUS were analyzed by two assistant chief physicians of ultrasound diagnosis. Area under the receiver operating characteristic curve analysis, sensitivity, specificity, positive predictive value, and negative predictive value were calculated to assess the diagnostic ability of different indices.

**Results:**

We found that the TDOA significantly distinguished benign inflammation from malignant lesions. The TDOA was markedly increased in patients with malignant lesions than benign inflammation lesions (*P* < 0.001). Compared with conventional time-intensity curve (TIC) indices, TDOA showed high diagnostic accuracy (area under the curve = 0.894). Moreover, conventional diagnostic indices did not affect the diagnostic performance of TDOA by adjusting the receiver operating characteristic curve.

**Conclusion:**

TDOA is feasible for the diagnosis of benign inflammation and malignant PPLs.

## Introduction

Lung cancer is one of the most common malignant tumors in the world with the highest incidence and mortality (1). According to the location of the tumor, lung cancer is divided into central type and peripheral type. Peripheral lung cancer accounts for about 70% of all lung cancer types and is difficult to be diagnosed with no obvious symptoms (2). The 5-year survival rate of lung cancer is about 70%–90% in the early stage (3–5), but approximately 75% patients are first diagnosed only in an advanced stage (6).

Common clinical methods such as dynamic contrast-enhanced magnetic resonance imaging (7), contrast-enhanced computed tomography (8, 9), low-dose computed tomography (10), and bronchoscopy (11) are used to diagnose the lung lesions. However, these methods have obvious diagnostic shortcomings, such as exposure to heavy dose of radiation (12), high cost (13, 14), and high false positive rates (15, 16). Some studies also have shown that the diagnostic capability of bronchoscopy is significantly reduced because of the increase in distance from the hilum and the small volume of tumor (17–19). Therefore, it is essential to improve the current screening and diagnostic ability.

Peripheral pulmonary lesions (PPLs) are close to the pleura, which can be easily detected by ultrasound. Contrast-enhanced ultrasound (CEUS) is a novel technique for real-time observation of disease states by using microbubble-based contrast agents (20, 21). CEUS has found application in many conditions such as myocardial inflammation (22), type I diabetes (23), carotid plaque (24), chronic kidney disease (25), and metastatic liver disease (9). A previous study has showed that the wash-in slope, time to peak intensity (TTP) and mean transit time (MTT) analyzed by CEUS can be used to distinguish the difference between malignant thoracic injuries and benign lesions (26). However, the low sensitivity and specificity of these conventional parameters for diagnosing PPLs by time intensity curves (TICs) analysis limits the application of CEUS.

In this study, we aimed to identify a novel index to diagnose the PPLs. For this purpose, we systematically analyzed the clinical data of 96 patients with PPLs and evaluated the efficacy of using time difference of arrival (TDOA) to distinguish between benign inflammation and malignant lesions.

## Materials and Methods

### Patients

**Figure 1:**
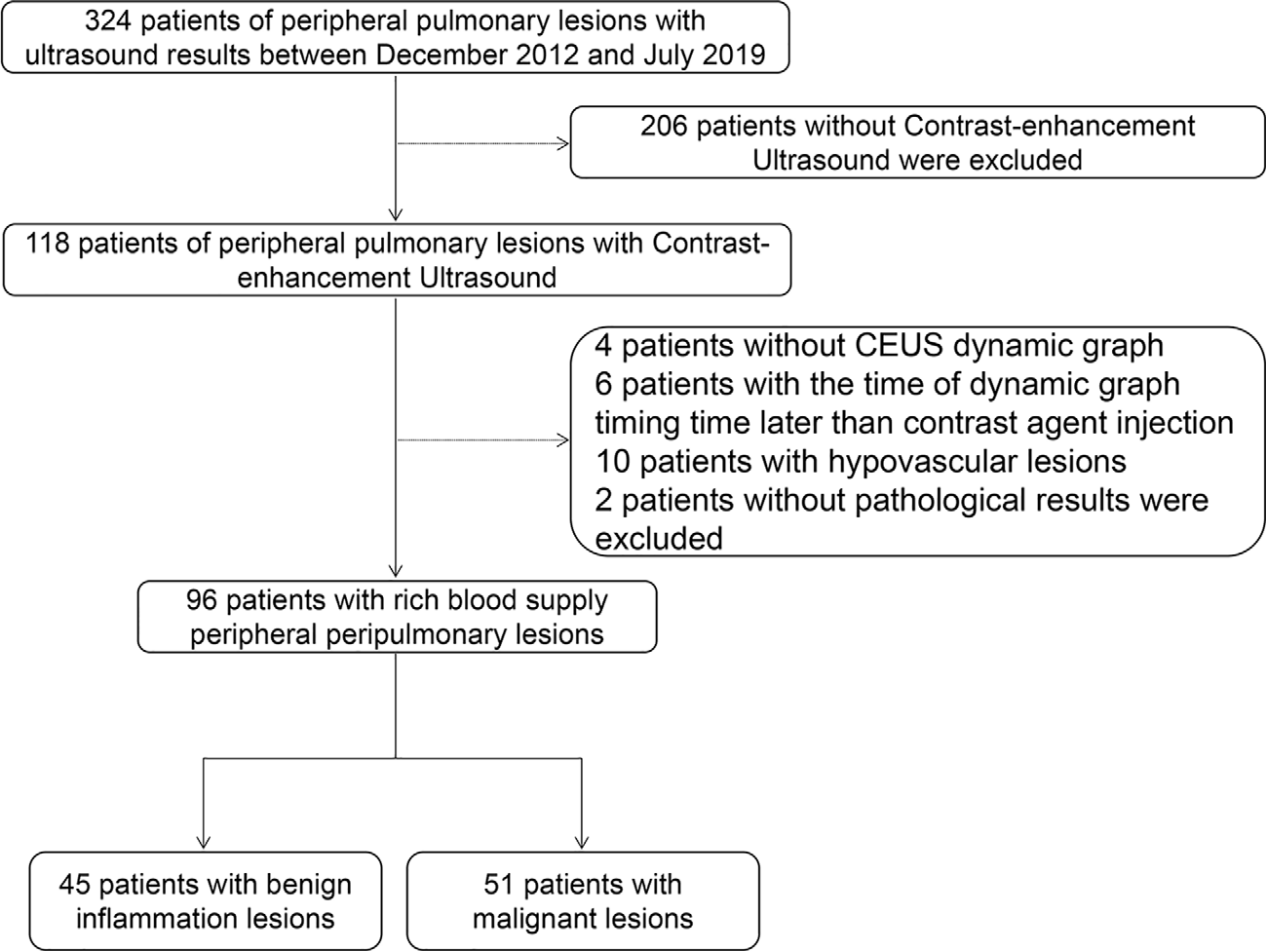
Workflow of the study. CEUS, contrast-enhanced ultrasound.

We retrospectively analyzed 324 patients with PPLs who had undergone CEUS and biopsy or histopathologic examination in our hospital between December 2012 and July 2019. The inclusion criteria were as follows: i) Age from 18 to 85 years old; ii) With contrast ultrasound and biopsy or histopathologic examination; iii) Under contrast conditions, all patients with peripulmonary lesions significantly enhanced after contrast-enhanced ultrasound compared with those without contrast medium injection were hypervascular lesions. The exclusion criteria were as follows: i) Patients with bleeding tendency, severe cardiac insufficiency, pulmonary hypertension, aortic aneurysm, mental disorders, and other causes of pulmonary insufficiency; ii) Patients allergic to contrast media, with poor physical condition and not cooperating with researchers. All patients were diagnosed by biopsy. 96 patients of them were included in this study and their ages ranged from 20 to 85 years (**Figure 1**, **Table 1**). Of the 96 patients, 45 had benign inflammation lesions: 37 (82.2%) and eight (17.8%) cases of nonspecific and specific inflammation, respectively. The remaining 51 patients had malignant lesions: 23 (45.1%) cases of squamous cell carcinoma and 21 (41.2%) of adenocarcinoma; the remaining seven (13.7%) patients had other malignancies. Patients were predominantly male (71.1% in the benign inflammation lesions group and 76.5% in the malignant lesions group). Left lung lesions accounted for 48.9% and 60.8% lesions in the benign inflammation and malignant lesions groups, respectively. There were no significant differences in demographic characteristics between the benign inflammation and malignant lesions groups (**Table 1**). The study was approved by the ethics committee of Dazhou Central Hospital (IRB00000003-19008).

**Table 1:** Characteristics of 96 patients with peripheral pulmonary lesions.

	Benign inflammation lesions (N = 45)	Malignant lesions (N = 51)
Male gender, n (%)	32 (71.1)	39 (76.5)
Age, year, mean (SE)	59.5 (2.18)	63.9 (1.01)
Left lung, n (%)	22 (48.9)	31 (60.8)
Smoking, n (%)		
Current/Former	26 (57.8)	33 (64.7)
Never	18 (40.0)	17 (33.3)
Unclearly	1 (2.22)	1 (1.96)
Alcohol consumption, n (%)		
Never	21 (46.7)	25 (49.0)
Frequently/Occasionally	22 (48.9)	25 (49.0)
Unclearly	2 (4.4)	1 (1.96)
Medical history, n (%)		
Hypertension	7 (15.6)	7 (13.7)
Diabetes	4 (8.89)	4 (7.84)
Pulmonary diseases	4 (8.89)	4 (7.84)
Malignant tumors	1 (2.22)	3 (5.88)
Pathologic types, n (%)		
Nonspecific inflammation	37 (82.2)	—
Specific inflammation	8 (17.8)	—
Squamous cell carcinoma	—	23 (45.1)
Adenocarcinoma	—	21 (41.2)
Other	0	7 (13.7)

SE, standard error. There was no significant difference among the groups (P > 0.05).

### Analysis of CEUS Parameters

A LOGIQ-E9 color Doppler ultrasonic diagnostic apparatus equipped with CEUS software, TIC analysis software, and a 3.5–5 MHz probe (GE Healthcare, Milwaukee, WI) were used in this study. The contrast agent for injection was white powder sulfur hexafluoride microbubble (SonoVue) for injection (Bracco SpA, Milan, Italy). Five milliliters physiological saline (0.9% NaCl) was added and agitated to form a microbubble suspension. We used conventional ultrasound to observe the size of the lesion, internal echo, and blood flow. Then, we placed the largest section of the lesion in the center of the display to initiate a double-contrast CEUS mode. Briefly, 2.4 mL sulfur hexafluoride microbubble suspensions were rapidly injected *via* the elbow vein, followed by a rapid bolus injection of 5 mL normal saline. The built-in timer of the ultrasound instrument was activated and observed for 3 min. All images were stored and analyzed.

Two assistant chief physicians with 5 years’ experience in ultrasound diagnosis who were blinded to the pathological diagnosis of the patient, observed the filling characteristics of the contrast agent in the lesion and performed the CEUS analysis together and reached an agreement. First, we observed the blood supply in the mass and the area where the large vessels were located, distinguished the active area from the necrotic area, traced the real part of the lesion and the adjacent lung tissue as the regions of interest (ROI), and ensured the same ROI in each depiction. Then, the TIC software that comes with the LOGIQ-E9 color Doppler ultrasound was used to quantitatively analyze the arrival time (AT) of the lesion and adjacent lung tissues. Next, we obtained the values TDOA (in triplicate) of time difference between the AT of the lesion and adjacent lung tissue (=lesion contrast agent AT-adjacent lung tissue contrast agent AT), TTP, initial intensity (II), peak intensity (PI), area under the curve (AUC), rake ratio (K), mean square error (MSE), and curve gradient and calculated the average. After the analysis, the receiver operating characteristic (ROC) curve of adjacent lung tissue, lesion contrast agent AT and TDOA were drawn. The Youden’s index was calculated to determine the best cut-off point of lesion AT and adjacent lung tissue time difference. If the arrival time was greater than the best cut-off point, the lesion was considered malignant; otherwise, it was considered benign inflammation.

### Puncture Biopsy

A physician with > 5 years’ experience performed the ultrasound biopsy of all patients. Infection markers, bleeding, and coagulation time of patients were required to be normal. The blood supply and large vessels in the mass were observed, and the puncture point was located in the area with blood supply but not large vessels. During the operation, the puncture point was fixed, the distance from the sampling point in the mass was measured, the puncture area was disinfected with iodine tincture, and the puncture point was anesthetized with 2% lidocaine for local infiltration. Next, under the guidance of contrast-enhanced ultrasound, an 18G automatic biopsy gun was used for subcutaneous penetration. The obtained specimens were fixed in 10% formaldehyde solution and sent for pathological examination.

### Statistical Analysis

Quantitative data were expressed as mean (standard error [SE]), and the difference between groups was analyzed by student’s *t*-test. The categorical data were expressed as numerical percentages. When theoretical frequency 1 ≤ N < 5, continuous correction chi-square test was used, and Pearson chi-square test was used for all theoretical frequencies n > 5. The sensitivity and specificity of the optimal cut-off point were calculated from the area under the ROC curve. The ROC curve constructed from logistic regression and the ROC-AUC difference was analyzed by the DeLong’s test. *P* < 0.05 was considered statistically significant. All the statistical methods were two tailed. The free degree of *t*-test for age, long diameter, and arrival time was 94, while the free degree of *t*-test for other parameters produced by the TIC analysis was 80.

## Results

### Ultrasound Characteristics

The mean long diameter was 5.54 cm for the benign inflammation lesions group and 7.71 cm for the malignant lesions group (*P* < 0.001). Benign inflammation lesions were mainly wedge shaped, while malignant lesions were mainly spherical (*P* < 0.001). However, the conventional two-dimensional ultrasound indicators were not specific enough for the differential diagnosis of benign inflammation and malignant lesions. In this study, the PI, II, TTP, AUC, K, MSE, and gradient values could only be obtained from 34 and 48 patients with benign inflammation and malignant lesions, respectively. Furthermore, we found that the perfusion method, TTP, and PI produced by CEUS also showed no difference between the benign inflammation and malignant lesions groups (*P* > 0.05) (**Table 2**).

**Table 2:** CEUS parameters of 96 patients with peripheral pulmonary lesions.

	Benign inflammation lesions (N = 45)	Malignant lesions (N = 51)	*P*-value
Two-dimensional ultrasound			
Long diameter (cm), mean (SE)	5.54 (0.26)	7.71 (0.40)	< 0.001
Lesion shape, n (%)			< 0.001
Wedge	26 (57.8)	4 (7.84)	
Irregular	14 (31.1)	20 (39.2)	
Spherical	5 (11.1)	27 (52.9)	
Bronchial tree sign, n (%)	7 (15.6)	6 (11.8)	0.59
CEUS			
Perfusion method, n (%)			0.22
Even perfusion	26 (57.8)	23 (45.1)	
Perfusion defect	19 (42.2)	28 (54.9)	
TIC analysis, mean (SE)			
Lesion AT (s)	5.54 (0.44)	8.69 (0.51)	< 0.001
Adjacent lung tissue AT (s)	4.45 (0.38)	4.65 (0.38)	0.71
Time difference of arrival (s)	1.09 (0.20)	4.03 (0.29)	< 0.001
Peak intensity (dB)^a^	23.8 (1.29)	22.3 (1.03)	0.34
Initial intensity (dB)^a^	-66.9 (4.30)	-66.8 (2.99)	0.99
Time to peak (s)^a^	17.5 (2.14)	14.5 (1.49)	0.24
Area under the curve^a^	1895 (88.9)	1802 (74.6)	0.42
Rake ratio^a^	4.50 (0.47)	4.59 (0.42)	0.88
Mean-square error^a^	9.41 (1.22)	7.81 (0.75)	0.24
Gradient^a^	1.89 (0.19)	2.05 (0.16)	0.52

a Only 34 benign inflammation lesion patients and 48 malignant lesion patients had the peak intensity, initial intensity, time to peak, area under the curve, rake ratio, mean-square error and gradient value.

CEUS, contrast-enhanced ultrasound; TIC, time-intensity curve; AT, arrival time.

### Diagnostic Performance Comparison of Arrival Time

**Figure 2:**
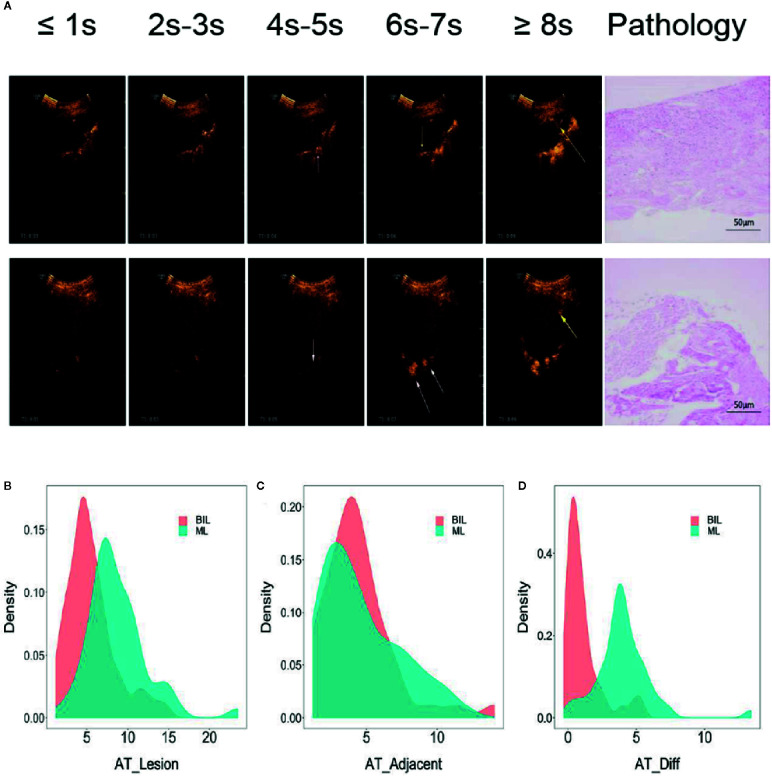
Typical images of contrast agent emerging and pathology of lung cancer and pneumonia. **(A)** The two left-most rows without arrows indicated that neither the lesion nor adjacent lung tissue is contrast-free. White arrows indicated that adjacent lung tissues begin to appear contrast agent, while yellow arrows indicated that the lesions appear contrast agent. The two right-most pictures were typical pathological images of various types. **(B–D)** Distribution of lesion AT, adjacent lung tissue AT, and time difference of arrival grouped by pathologic types. Benign inflammation lesions group (N = 45), malignant lesions group (N = 51), Student’s *t*-test. BIL, benign inflammation lesions; ML, malignant lesions; AT, arrival time; Diff, difference.

**Figure 3:**
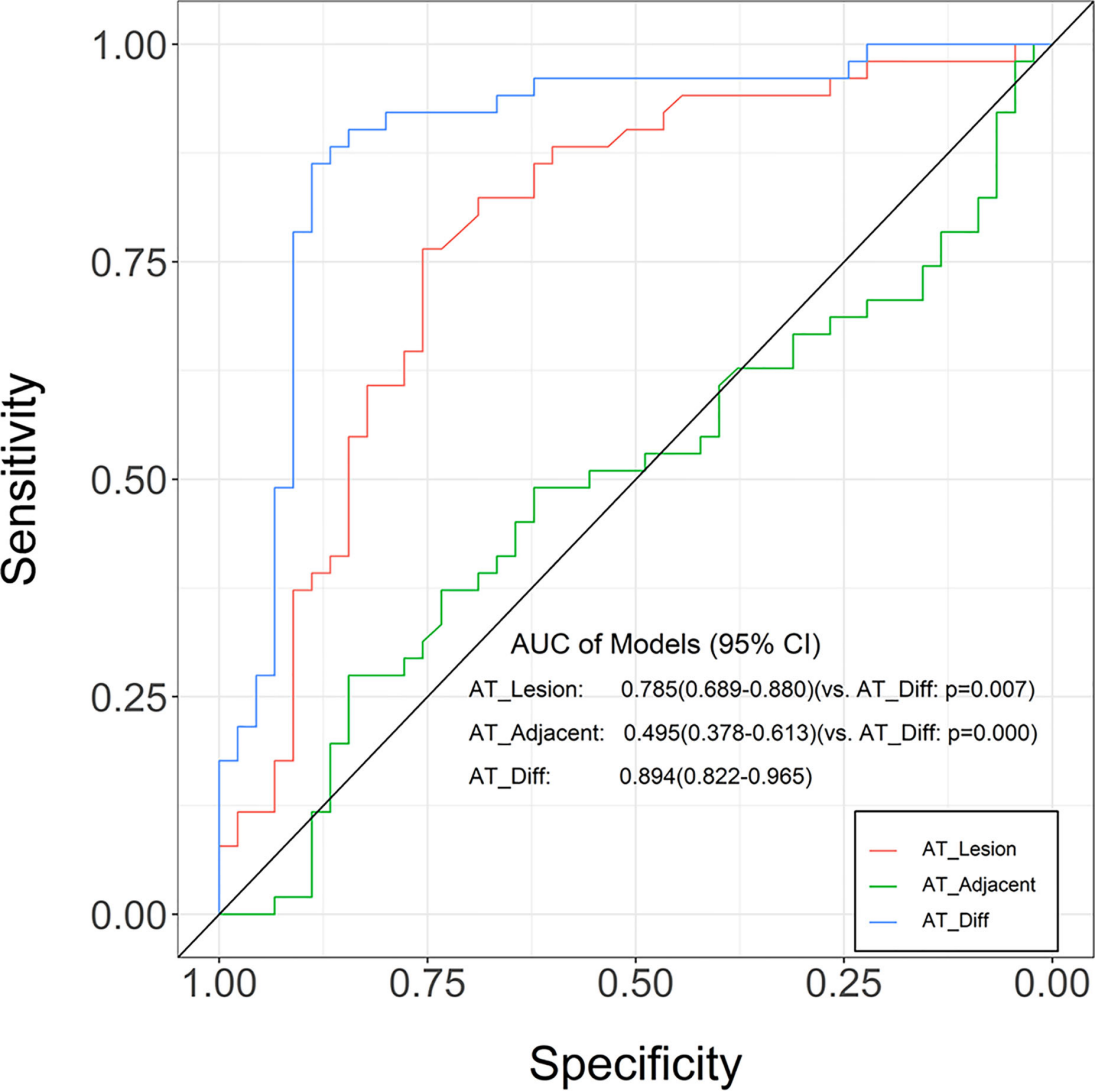
Receiver operating characteristic (ROC) curves for the lung tissue AT, lesion AT, and time difference of arrival in distinguishing between benign inflammation and malignant lesions. Compared with adjacent lung tissue AT and lesion AT, the time difference of arrival was greater in AUC. The ROC curve is expressed by diagnostic sensitivity and specificity. AT, arrival time; Diff, difference.

Based on TIC analysis, we generated the lesion AT, adjacent lung tissue AT, and a novel method of TDOA. The lesion AT and TDOA in the benign inflammation lesions group was shorter than those of the malignant lesions group (*P* < 0.001), but there was no difference in the adjacent lung tissue AT between both groups (**Figure 2** and **Figure S1**). TDOA had the largest area under the curve (AUC, 0.894), compared with the lesion arrival time (AUC, 0.785) and adjacent lung tissue arrival time (AUC, 0.495) (**Figure 3**). The optimal cut-off value of TDOA was 2.42 s. The sensitivity and specificity of TDOA were 86.3% (95% CI: 76.5–94.1) and 88.9% (95% CI: 80.0–97.8), respectively. Positive predictive value (PPV) and negative predictive value (NPV) were 85.1% (95% CI: 71.7–93.8) and 89.8% (95% CI: 77.8–96.6), respectively (**Table 3**). The PPV and NPV of TDOA were higher than the lesion AT and adjacent lung tissue AT.

**Table 3:** Diagnostic performance of lesion AT, adjacent lung tissue AT, and time difference of arrival in benign inflammation lesions and malignant lesions group.

Items	AT_Lesion	AT_Adjacent	AT_Diff
AUC	0.785 (0.689–0.880)	0.495 (0.378–0.613)	0.894 (0.822–0.965)
Cut-off (s)	6.55	2.48	2.42
Sensitivity (%) (95% CI)	76.5 (64.7–86.3)	27.5 (15.7–39.2)	86.3 (76.5–94.1)
Specificity (%) (95% CI)	75.6 (64.4–88.9)	84.4 (73.3–95.6)	88.9 (80.0–97.8)
PPV (%) (95% CI)	73.9 (58.9–85.7)	33.3 (14.6–57.0)	85.1 (71.7–93.8)
NPV (%) (95% CI)	78.0 (64.0–88.5)	49.3 (37.6–61.1)	89.8 (77.8–96.6)

AUC, area under the curve; PPV, positive predictive value; NPV, negative predictive value; CI, confid-ence interval; AT, arrival time; Diff, difference.

### ROC Curve of TDOA Adjusted by the Index of Patients

**Figure 4:**
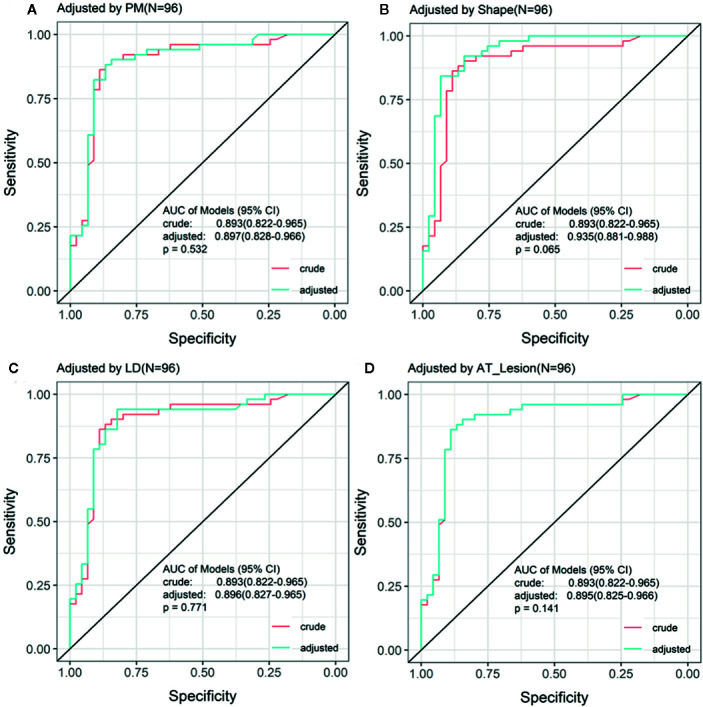
Receiving operating characteristic (ROC) curve adjusted by the indices of patients. The ROC curve adjusted by the top four different indices for patients. There was no significant difference between crude ROC and adjusted ROC curve. AT, arrival time; PM, perfusion method.

To evaluate whether other CEUS parameters or clinical information affected the prediction performance of TDOA, we used different indicators to adjust the ROC curve. The adjusted ROC curve showed that other indicators such as age or TTP did not affect the diagnostic performance of TDOA (**Figure 4** and **Figure S2**). Next, we investigated the role of AT in the subgroup analysis. The results showed that the lesion AT and adjacent lung tissue AT could not distinguish between non-specific and specific inflammation. Significantly, TDOA could differentiate between the two groups well (**Figure S3**). However, all AT parameters did not distinguish between squamous cell carcinoma and adenocarcinoma (**Figure S4**).

## Discussion

Although many imaging technologies have been used to diagnose the PPLs, there are considerable limitations, especially in terms of diagnostic capability (17), cost (13), and radiation exposure (12), which pose challenges to PPLs diagnosis. In this study, we identified a novel method for the differential diagnosis of benign inflammation and malignant PPLs using TDOA produced by CEUS. Our results showed that the TDOA was significantly increased in the malignant lesions group than the benign inflammation lesions group. Notably, conventional diagnostic parameters did not affect the diagnostic performance of TDOA.

Previous study has indicated that CEUS divides the arterial phases into pulmonary and bronchial in the PPLs, and the pulmonary artery phase is slightly earlier than the bronchial artery phase (27). In this study, we found that the TDOA of the malignant lesions group (4.03 s) was significantly longer than the benign inflammation lesions group (1.09 s). This finding is supported by previous studies that showed that the blood supply of malignant tumors mainly originates from the bronchial artery, while the blood supply of non-neoplastic pulmonary lesions comes from the pulmonary and bronchial arteries (28).

A large body of evidence has debated the value of CEUS in diagnosing lung diseases (29, 30). We found that TDOA produced by CEUS could well distinguish between benign inflammation and malignant lesions (AUC: 0.894). In addition, compared to the lesion AT, the TDOA excluded the influence of the patient’s heart function (31), blood supply status of the lesion area, and injection rate of the contrast agent (26). In our study, the TDOA had a higher sensitivity and specificity than the lesion AT and the adjacent lung tissue AT. Based on TIC analysis, we generated many parameters such as TTP. No significant differences were found between the benign inflammation and malignant lesions group (**Table 2**). Our findings are distinct from previously reported data on the role of TTP (26). These results suggested that the diagnostic performance of TDOA was better than the conventional parameters. Furthermore, our results showed that there was no significant difference between the crude and adjusted ROC curve, indicating that other indicators did not affect the diagnostic performance of TDOA.

Our study has some limitations. First, it was a single-center study and the number of patients was relatively small. Second, the major type of benign lesions were inflammation. More pathologic types of benign lesions are required to validate the applicability of TDOA. Last, it was a retrospective study.

## Conclusions

We systemically analyzed and validated TDOA produced by CEUS to distinguish between benign inflammation and malignant PPLs with high diagnostic accuracy. These findings showed that the TDOA could be a feasible, sensitive, and specific method to diagnose PPLs.

## Data Availability Statement

The raw data supporting the conclusions of this article will be made available by the authors, without undue reservation.

## Ethics Statement

The study was approved by the Ethics Committee of Dazhou Central Hospital (IRB00000003-19008). The Ethics Committee waived the need for patients to sign informed consent.

## Author Contributions

FXZ designed the study, revised the manuscript, and the figure. MT analyzed the CEUS parameters. QX drafted the manuscript and analyzed the data. JW analyzed the data. XYZ and JC revised the manuscript. HL collected the clinical characteristics and information. XXZ collected the clinical characteristics and information. GW analyzed the TICs. YT analyzed the TICs. FWZ organized the basic data. YC revised the manuscript and organized the basic data. JS recruited patients with PPLs and collected the clinical characteristics and information. All authors contributed to the article and approved the submitted version.

## Funding

This study was funded by the National Natural Science Foundation of China (81902861), “Xinglin Scholars” Scientific Research Project Fund of Chengdu university of traditional Chinese medicine (YYZX2019012), the Scientific Research Fund of Technology Bureau in Dazhou (17YYJC0004, 17YYJC0001), Key Research and Development Project Fund of Science and Technology Bureau in Dazhou, Sichuan Province (20ZDYF0001).

## Conflict of Interest

The authors declare that the research was conducted in the absence of any commercial or financial relationships that could be construed as a potential conflict of interest.
