# Supplementary material for: Time Difference of Arrival on Contrast-Enhanced Ultrasound in Distinguishing Benign Inflammation From Malignant Peripheral Pulmonary Lesions
Source: Front Oncol. 2020 Nov 12;10:578884. doi: 10.3389/fonc.2020.578884 (PMC7689010; doi:10.3389/fonc.2020.578884)
Supplement: Supplementary file 1 [file DataSheet_1.pdf]

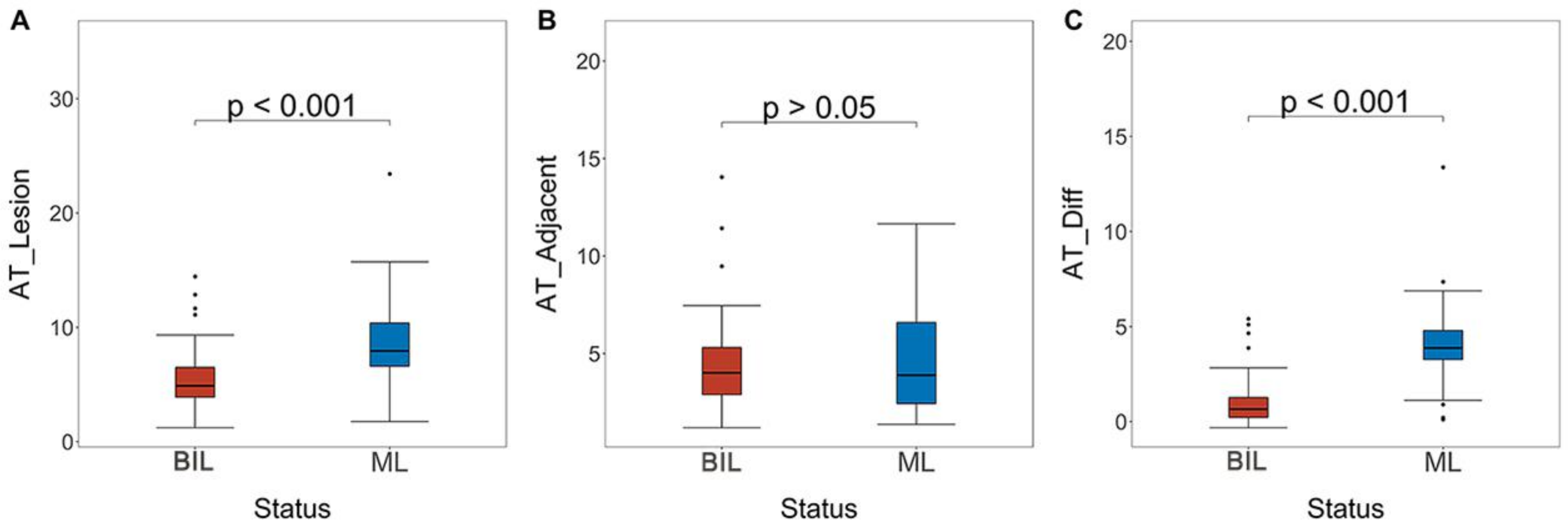

Figure S1. Arrival time in diagnosis of BIL and ML.
(**A-C**) Boxplot of lesion AT, adjacent lung tissue AT and time difference of arrival in BIL and ML group. BIL group N=45, ML group N=51, student's *t* test. BIL, benign inflammation lesions; ML, malignant lesions; AT, arrival time; Diff, difference.

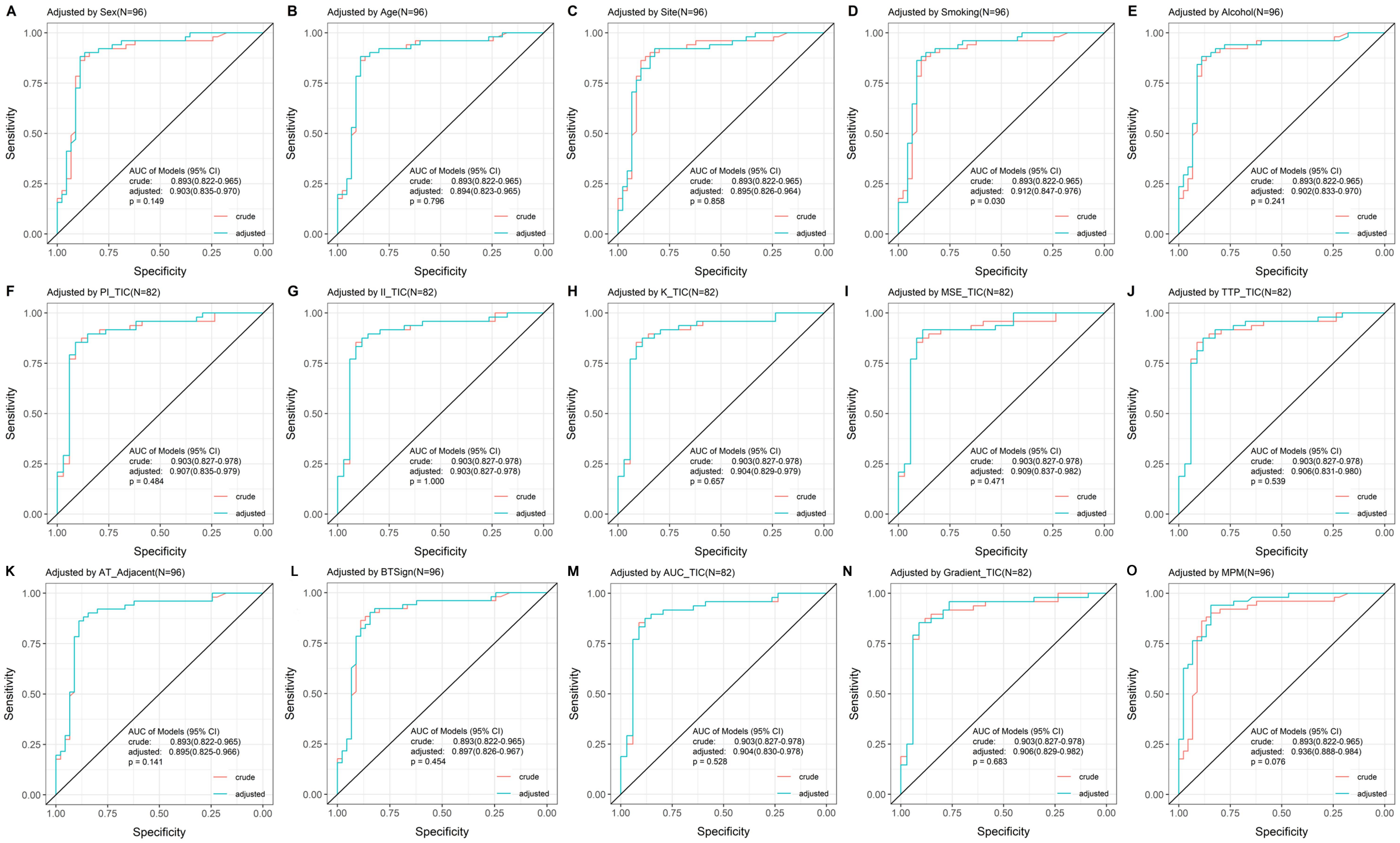

Figure S2. Receiving operator characteristic curve adjusted by the remained indices of patients. The ROC curve adjusted by the remained indices of patients. There was no significant difference between crude ROC and adjusted ROC curve. PI, peak intensity; TIC, time-intensity curve; II, initial intensity; K, rake ratio; MSE, mean-square error; TTP, time to peak; AT, arrival time; Btsign, bronchial tree sign; AUC, area under the curve; MPM, microcirculation perfusion mode.

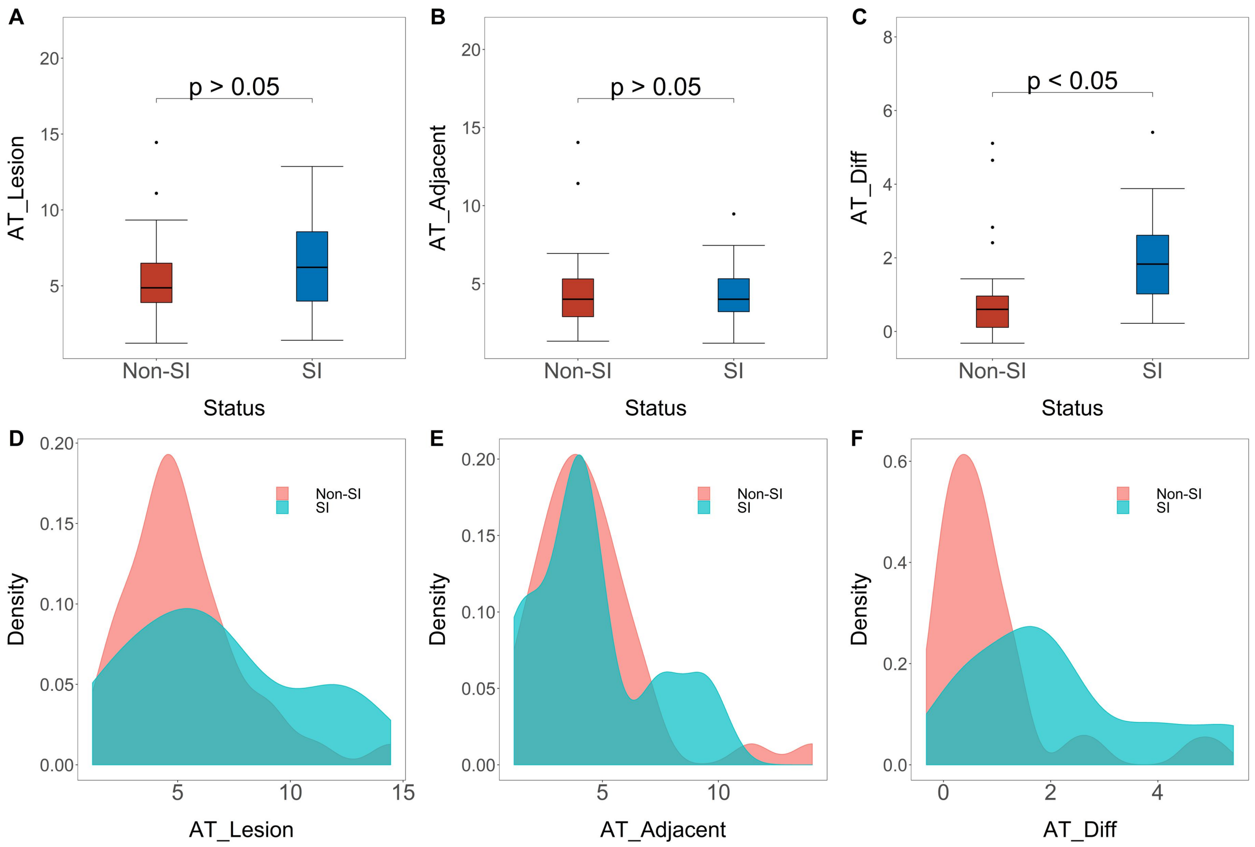

Figure S3. Arrival time in diagnosis of Non-SI and SI.
(**A-C**) Boxplot of lesion AT, adjacent lung tissue AT and time difference of arrival in Non-SI and SI group. (**D-F**) Distribution of lesion AT, adjacent lung tissue AT and time difference of arrival grouped by pathologic types. Non-SI group N=37, SI group N=8, student's *t* test. Non-SI, nonspecific inflammation; SI, specific inflammation; AT, arrival time; Diff, difference.

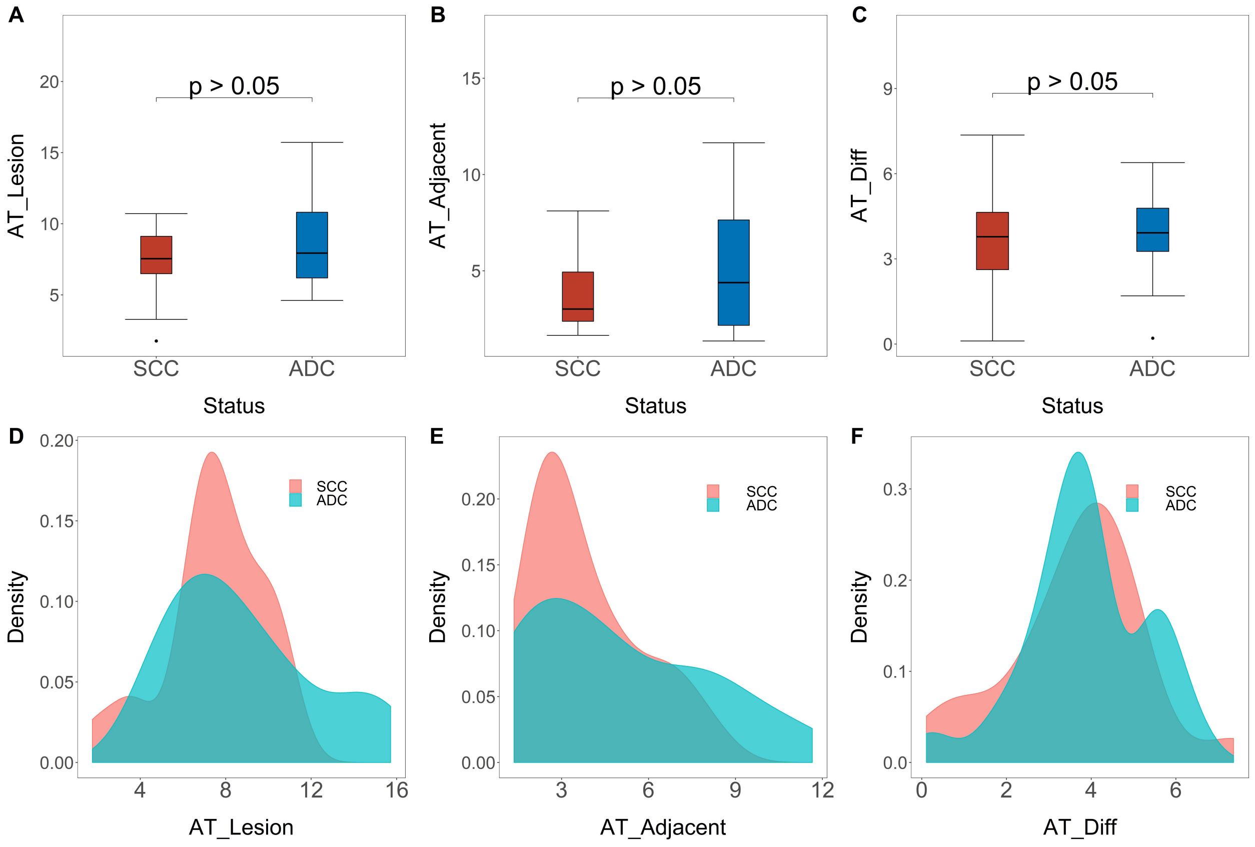

Figure S4. Arrival time in diagnosis of SCC and ADC.
(**A-C**) Boxplot of lesion AT, adjacent lung tissue AT and time difference of arrival in SSC and ADC group. (**D-F**) Distribution of lesion AT, adjacent lung tissue AT and time difference of arrival grouped by pathologic types. SCC group N=21, ADC group N=23, student's *t* test. SCC, squamous cell carcinoma; ADC, adenocarcinoma; AT, arrival time; Diff, difference.
